# Predisposition to Alcohol Drinking and Alcohol Consumption Alter Expression of Calcitonin Gene-Related Peptide, Neuropeptide Y, and Microglia in Bed Nucleus of Stria Terminalis in a Subnucleus-Specific Manner

**DOI:** 10.3389/fncel.2019.00158

**Published:** 2019-04-30

**Authors:** Ilaria Rossetti, Laura Zambusi, Paola Maccioni, Roberta Sau, Luciano Provini, M. Paola Castelli, Krzysztof Gonciarz, Giancarlo Colombo, Stefano Morara

**Affiliations:** ^1^Institute of Neuroscience, National Research Council of Italy, Milan, Italy; ^2^Department of Biotechnology and Translational Medicine, University of Milan, Milan, Italy; ^3^Department of Biomedical Sciences, Division of Neuroscience and Clinical Pharmacology, University of Cagliari, Cagliari, Italy; ^4^Center for Systems Biology Dresden, Max Planck Institute of Molecular Cell Biology and Genetics, Dresden, Germany

**Keywords:** BNST, CGRP, NPY, microglia, alcohol consumption, anterior subnuclei, Sardinian alcohol-preferring rats, Sardinian alcohol-non preferring rats

## Abstract

Excessive alcohol consumption is often linked to anxiety states and has a major relay center in the anterior part of bed nucleus of stria terminalis (BNST). We analyzed the impact of (i) genetic predisposition to high alcohol preference and consumption, and (ii) alcohol intake on anterior BNST, namely anterolateral (AL), anteromedial (AM), and anteroventral (lateral + medial subdivisions: AVl, AVm) subnuclei. We used two rat lines selectively bred for low- and high-alcohol preference and consumption, named Sardinian alcohol-non preferring (sNP) and -preferring (sP), respectively, the latter showing also inherent anxiety-related behaviors. We analyzed the modulation of calcitonin gene-related peptide (CGRP; exerting anxiogenic effects in BNST), neuropeptide Y (NPY; exerting mainly anxiolytic effects), and microglia activation (neuroinflammation marker, thought to increase anxiety). Calcitonin gene-related peptide-immunofluorescent fibers/terminals did not differ between alcohol-naive sP and sNP rats. Fiber/terminal NPY-immunofluorescent intensity was lower in BNST-AM and BNST-AVm of alcohol-naive sP rats. Activation of microglia (revealed by morphological analysis) was decreased in BNST-AM and increased in BNST-AVm of alcohol-naive sP rats. Prolonged (30 consecutive days), voluntary alcohol intake under the homecage 2-bottle “alcohol vs. water” regimen strongly increased CGRP intensity in BNST of sP rats in a subnucleus-specific manner: in BNST-AL, BNST-AVm, and BNST-AM. CGRP area sum, however, decreased in BNST-AM, without changes in other subnuclei. Alcohol consumption increased NPY expression, in a subnucleus-specific manner, in BNST-AL, BNST-AVl, and BNST-AVm. Alcohol consumption increased many size/shapes parameters in microglial cells, indicative of microglia de-activation. Finally, microglia density was increased in ventral anterior BNST (BNST-AVl, BNST-AVm) by alcohol consumption. In conclusion, genetic predisposition of sP rats to high alcohol intake could be in part mediated by anterior BNST subnuclei showing lower NPY expression and differential microglia activation. Alcohol intake in sP rats produced complex subnucleus-specific changes in BNST, affecting CGRP/NPY expression and microglia and leading to hypothesize that these changes might contribute to the anxiolytic effects of voluntarily consumed alcohol repeatedly observed in sP rats.

## Introduction

Excessive alcohol consumption poses a serious threat to human health. This can arise, for example, from increased risk of cancer or cardiovascular diseases (Kunzmann et al., 2018; Wood et al., 2018). A major threat, however, arises also from behavioral and neurobiological effects produced by excessive alcohol drinking, in particular binge drinking, by young people (but also adults; Kranzler and Soyka, 2018). Since youth represents a critical period in brain development that is particularly vulnerable to alcohol misuse, binge/heavy-drinking during adolescence and in young adults carries major long-term consequences. Indeed, binge and heavy-drinking adolescents and young adults have altered neural structure and activity which could result in neural reorganization and increased risk for developing alcohol use disorder (Cservenka and Brumback, 2017) and can develop long-term cognitive and psychological consequences (Carbia et al., 2018). Hence, alcohol drinking deserves careful analysis and has been indeed the focus of several studies to identify brain areas and mechanisms driving consumption motivations.

One of such brain areas is bed nucleus of stria terminalis (BNST). BNST is, together with central and medial amygdala, a main part of the so-called extended amygdala whose glutamatergic and GABAergic heterogeneous populations of neurons are thought to determine aversive and appetitive behaviors, respectively (Waraczynski, 2016). In particular, BNST has been involved in several alcohol-mediated effects (Wills and Winder, 2013), in behaviors related to drugs of abuse (including cocaine, nicotine, alcohol, morphine; Vranjkovic et al., 2017), in increased drinking behavior associated with chronic alcohol exposure and withdrawal (as regulator of anxiety; Kash, 2012), and its intrinsic anxiogenic and anxiolytic circuits can be differentially modulated by specific drugs of abuse (e.g., cocaine, alcohol; Daniel and Rainnie, 2016). Alcohol consumption is frequently related to anxiety-related behaviors and in BNST circuitry anxiety-dependent physiological activation has been shown to be processed in a complex manner (Gungor and Paré, 2016; Goode and Maren, 2017; Luyck et al., 2018).

Calcitonin gene-related peptide (CGRP) signaling system is abundantly expressed in BNST: CGRP-immunoreactive fibers and terminals, derived from parabrachial nucleus, are distributed in several subnuclei (Dobolyi et al., 2005), and CGRP receptors have been detected by autoradiography (Skofitsch and Jacobowitz, 1985). In BNST-AL CGRP application potentiates anxiety-like behaviors by inhibiting GABAergic projection neurons (Gungor and Paré, 2014). However, both anxiogenic and anxiolytic effects have been ascribed to BNST, in particular to ventral BNST (vBNST; see below) upon stimulation of its glutamatergic and GABAergic neurons, respectively (Jennings et al., 2013: the boundaries of vBNST considered by these authors include what here will be referred to as BNST-AVl and part of BNST-AVm). BNST-AVl contains moderate/high levels of CGRP fibers and terminals (Dobolyi et al., 2005) and a large proportion of CGRP synapses in BNST are established on GABAergic neurons (see, e.g., Kozicz and Arimura, 2001). It is, thus, likely that CGRP action in BNST could be more complex than merely anxiogenic.

Other molecular mechanisms have been described, so far, to underlie the processing of anxiety-related and appetitive/aversive behaviors in BNST, that include NPY (but also CRF) signaling (see, e.g., Pleil et al., 2015). As a whole, NPY is thought to exert an anxiolytic role, although postsynaptic Y1 receptor signaling exerts potent anxiolytic effects whereas presynaptic Y2 receptors augment anxiety (see Tasan et al., 2010 and references therein). Its actions are, however, more complex. In BNST, NPY is present in fibers/terminals that originate from central amygdala (Wood et al., 2016), but also from other nuclei (see, e.g., Riche et al., 1990). NPY signaling can originate through direct release from BNST afferents, but also dendritic release (Ludwig and Leng, 2006) from scattered BNST NPY-positive cells (Wood et al., 2016; our unpublished observations). Decreased NPY signaling might be causally linked to depression: this was seen in a model of type 2 diabetes in extended amygdala, including ventral part of BNST lateral division (here named BNST-AVl; Nakhate et al., 2016) as well as in CCK-4-induced anxiety- and depression-like behaviors in limbic areas, including ventral part of BNST lateral division (here named BNST-AVl; Desai et al., 2014). In a conditioned place aversion test, it was found that intra-dl BNST (here named BNST-AL) NPY injection suppressed pain-induced aversion (an effect mediated by Y1/Y5 receptors; Ide et al., 2013). NPY Y2 receptors in anteroventral BNST (BNST-AV) facilitates fear extinction and attenuates retrieval of remote fear independently of extinction training (Verma et al., 2018). However, puzzling results have been found on anxiety. A correlation was found between decrease of Y2 receptor density in stria terminalis/BNST (and central amygdala) and decrease in anxiety- (and depression-like) behavior following its ablation in central amygdala (Y2 receptor is predominantly presynaptic; Tasan et al., 2010): on the other side, bilateral BNST infusion of a Y2 receptor antagonist had no measurable effects on anxiety-like or locomotor behavior in a binge alcohol drinking model (Pleil et al., 2015). In this last model, Y1 receptor activation in the BNST suppressed binge alcohol drinking without alteration in anxiety-like or locomotor behavior in the open field test, suggesting that the behavioral effects of Y1 receptor manipulation were specific to binge alcohol drinking. Manipulation of BNST Y2 receptor may thus alter general reward-seeking/appetitive behaviors, whereas binge alcohol drinking seems to be modulated by Y1 receptor (Pleil et al., 2015).

Another process which is thought to be involved in anxiety-related behaviors is neuroinflammation, which is emerging as a crucial factor in anxiety in humans and anxiety-related behaviors in animal models under a variety of experimental conditions (see, e.g., Liu et al., 2018; Nie et al., 2018; Zhang et al., 2018).

Since CGRP and NPY can potently modulate neuroinflammation (Ferreira et al., 2012; Gonçalves et al., 2012; Sardi et al., 2014; Morara et al., 2015; Rossetti et al., 2018) and NPY potently regulates alcohol consumption (Pleil et al., 2015), we here analyzed if and how CGRP, NPY, and neuroinflammation (revealed by means of morphological microglia activation: Aguzzi et al., 2013) in BNST are differentially regulated (i) in rat lines selectively bred for opposite alcohol preference and consumption and (ii) by voluntarily consumed alcohol in alcohol-preferring rats. To address these two research questions, we used the Sardinian alcohol-preferring (sP) and -non preferring (sNP) rat lines, one of the few pairs of rat lines selectively bred worldwide for high and low alcohol preference and consumption, respectively (see Colombo et al., 2006). When given a choice between 10% (v/v) alcohol and water under the standard, homecage 2-bottle regimen, with unlimited access for 24 h/day, sP rats display a clear preference for the alcohol solution and consume daily 6–7 g/kg pure alcohol (Colombo et al., 2006). Voluntary alcohol intake in sP rats gives rise to relatively high blood alcohol levels and produces measurable psychopharmacological effects, including locomotor stimulation and amelioration of genetically based anxiety-related behaviors (Colombo et al., 2006). Notably, sP rats meet all the fundamental requirements posed when defining an animal model of alcohol use disorder (Colombo et al., 2006). Conversely, sNP rats avoid alcohol virtually completely, even when exposed for relatively long periods to mixtures containing alcohol and palatable tastants (Colombo et al., 2006).

## Materials and Methods

### Animals

Adult, male sP and sNP rats (aged 60 days at the start of the experiment) from the 101^st^ generation were used. Animals were individually housed in standard plastic cages with wood chip bedding. The animal facility was under an inverted 12:12 h light–dark cycle (lights on at 9:00 p.m.), at a constant temperature of 22 ± 2°C and relative humidity of approximately 60%. Rats had free access to standard rat chow throughout the experimental period. Rats were divided into three groups: alcohol-naive sP (*n* = 10), alcohol-naive sNP (*n* = 10), and alcohol-experienced sP (*n* = 10) rats. Alcohol-naive rats had free access to tap water throughout the experimental period. Starting from the age of 60 days, alcohol-experienced sP rats were exposed to the standard, homecage 2-bottle choice regimen between an alcohol solution (10% in tap water, v/v) and tap water with unlimited access (24 h/day) for 30 consecutive days. The left-right position of the two bottles was randomly interchanged to avoid the development of position preference. Alcohol and water intake were recorded once daily (before the start of the dark phase) by weighing the bottles (0.1-g accuracy).

### BNST Analysis

Rats were sacrificed by perfusion with 4% paraformaldehyde following deep general anesthesia with equithesin (3 ml/kg i.p.; 2.1 g chloral hydrate, 0.46 g sodium pentobarbital, 1.06 g MgSO_4_, 21.4 ml propylene glycol, 5.7 ml ethanol (90%), 3 ml H_2_O). Briefly, following perfusion (30 min) the brains were embedded in sucrose, frozen, cut at cryostat in the coronal plane at 10 μm thickness and collected on gelatin-coated slides as previously described (Morara et al., 2000, 2001). In particular, parallel series of adjacent sections were collected on different slides with cycles of 500 μm, i.e., each section (within a slide) was 500 μm apart from adjacent ones. Subsequently brain sections were processed for histology and immunofluorescence as previously described (Rossetti et al., 2018). A Nissl stain was used to identify rostro-caudal level of sections (following coordinates described in “The rat brain in stereotaxic coordinates,” Paxinos and Watson, 1986).

The following primary antisera/antibodies or binding ligand were used: CGRP (1:1000, rabbit polyclonal, Peninsula, T-4032), GFAP (1:1500, monoclonal, Sigma, G3893), NPY (1:1000, rabbit polyclonal, Sigma, N9528), DyLight594-conjugated Tomato (Lycopersicon Esculentum) Lectin (TL; 1:200, Vector, DL-1177). Secondary antisera included Alexa-Fluor 488-conjugated donkey anti-rabbit Affinity purified (1:400, Molecular Probes, A21206), Alexa-Fluor 594-conjugated donkey anti-mouse Affinity purified (1:200, Molecular Probes, A21203). DAPI staining was used to label nuclei.

The labeled sections were analyzed by LSM 800 confocal Zeiss microscope and single sections or Z-scan series were taken with oil immersion 40 X/NA 1.30 objective. Z-scan series were taken at 1.0 μm interval. The images (single sections or Z-scan series) were initially processed by background subtraction: this was achieved by MosaicSuite free software^1^ for ImageJ.^2^ In our hands this software provided better results than other ImageJ background subtraction method (e.g., Rolling Ball) as produced reduced smoothing effect while obtaining same background threshold level (not shown). The cleaned (background-subtracted) images were then segmented by Moments automatic ImageJ thresholding methods, normalized (using the median levels of background of images taken from alcohol-naive sNP rats as reference) and automatically analyzed by a home-made macro in ImageJ. Exclusion of unwanted tissue areas (e.g., lateral ventricle spaces, wrinkles) was performed manually.

The analysis of neuropeptide expression was focused on BNST subnuclei that received the main CGRP and NPY innervation, anterolateral (AL), anteromedial (AM) and anteroventral (AV, medial and lateral) subnuclei (see Gungor and Paré, 2016: here, AV subnucleus was divided in medial, AVm, and lateral, AVl). Theoretically, changes of neuropeptide innervation could be driven by changes in intensity (i.e., changes in neuropeptide content) within individual fiber/terminals, size of labeled structures or total number of labeled structures (measured as sum of areas): in order to monitor these types of changes, the analysis comprised measurement of intensity (median fluorescence intensity), mean labeled area (area of each labeled particle) and sum of labeled areas (which can be dependent also on their number). Immunofluorescence of CGRP- or NPY-labeled fibers/terminals can strongly vary throughout the rostro-caudal extent of each BNST subnucleus: changes in total number of labeled structures were monitored by analyzing sum of labeled areas in each section by taking into account the rostro-caudal level of the section. To this aim, the analysis was performed via the following steps: (1) 3–4 scans (from 2 to 3 sections) at each of three rostro-caudal levels for BNST-AM and BNST-AVm (+0.35, -0.05, -0.45 mm from Bregma) or two levels for BNST-AL, BNST-AVl (-0.05, -0.45 mm from Bregma), (2) calculation of the sum of labeled areas in each section, (3) calculation of the median values of the sum of areas at each rostro-caudal level (for each subnucleus), (4) calculation of the sum of the median values of the two/three rostro-caudal levels (for each subnucleus). A parallel analysis showed that mean area of neuropeptide-labeled structures did not change, and hence expression changes were found to be only dependent on changes in number of labeled fibers/terminals and fluorescence intensity (not shown).

Morphological analysis of microglia was performed on Tomato Lectin-stained cells that showed also DAPI-staining: Tomato Lectin-labeled vessels were deleted manually. The analysis was performed by measuring automatically (via a home-made ImageJ macro) the following parameters: median, area, perimeter, height, width (height and width are the dimensions of the smallest rectangle enclosing the selected particle), major, minor (major and minor are the primary and secondary axis of the best ellipse fitting the selected particle), minferet (the smallest distance between two parallel tangents touching the particle outline in all directions, also known as minimum caliper) and aspect ratio (indicated as AR: with a value of 1.0 indicating a perfect circle, while as the value approaches 0.0, it indicates an increasingly elongated shape).

An indirect analysis of microglia proliferation was finally performed by measuring density of microglia cells that showed nuclear labeling by DAPI. After manual removal of Tomato Lectin-labeled vessels, a home-made macro identified a labeled cell based on the presence of Tomato lectin-labeling in a thin rim around DAPI-stained nucleus (Sardi et al., 2014). Microglia cells were then automatically counted.

### Statistics

All analyses were performed using the GraphPad Prism software. Data on daily alcohol intake in alcohol-experienced sP rats were analyzed by a 1-way ANOVA with repeated measures. In each experimental session of immunofluorescence, expression levels were calculated as percentage of values detected in alcohol-naive sNP rats. Comparisons were made between alcohol-naive sNP and sP rats, as well as between alcohol-naive and -experienced sP rats. The tests were two-tailed. Distributions of continuous variables showing departure from normality was assessed by Shapiro-Wilk test. The analysis was then performed by ANOVA (*F* statistic; Bonferroni correction was considered for multiple comparisons) when variable distribution was found to follow normality, or non-parametric Kruskal-Wallis test (*H* statistic; Dunn’s multiple comparison test) when variable distribution was found to deviate from normality. ANOVA (*F*) or Kruskal-Wallis (*H*) statistics were initially reported as analysis of the three groups: then, comparisons of specific experimental couples by Bonferroni or Dunn’s multiple tests were also indicated.

## Results

### CGRP Expression

Comparisons of CGRP expression were made between (i) alcohol-naive sNP and sP rats, with the intent of assessing if and how genetic predisposition to alcohol preference and consumption impacted on CGRP expression, and (ii) alcohol-naive and -experienced sP rats, with the intent of assessing the impact of voluntary consumption of pharmacologically relevant amounts of alcohol on CGRP expression. At first, an overall analysis of the three groups was carried out: this showed a significant difference in CGRP content (in particular intensity) among the three groups in BNST-AL (CGRP intensity: *F* = 27.02, *p* < 0.0001; CGRP area sum: *F* = 0.8413, *p* = 0.4435), BNST-AM (CGRP intensity: *F* = 54.96, *p* < 0.0001; CGRP area sum: *H* = 14.11, *p* = 0.0009) and BNST-AVm (CGRP intensity: *F* = 11.84, *p* = 0.0002; CGRP area sum: *H* = 9.190, *p* = 0.0101), but not in BNST-AVl (CGRP intensity: *H* = 1.071, *p* = 0.5854; CGRP area sum: *H* = 1.554, *p* = 0.4597).

**FIGURE 1:**
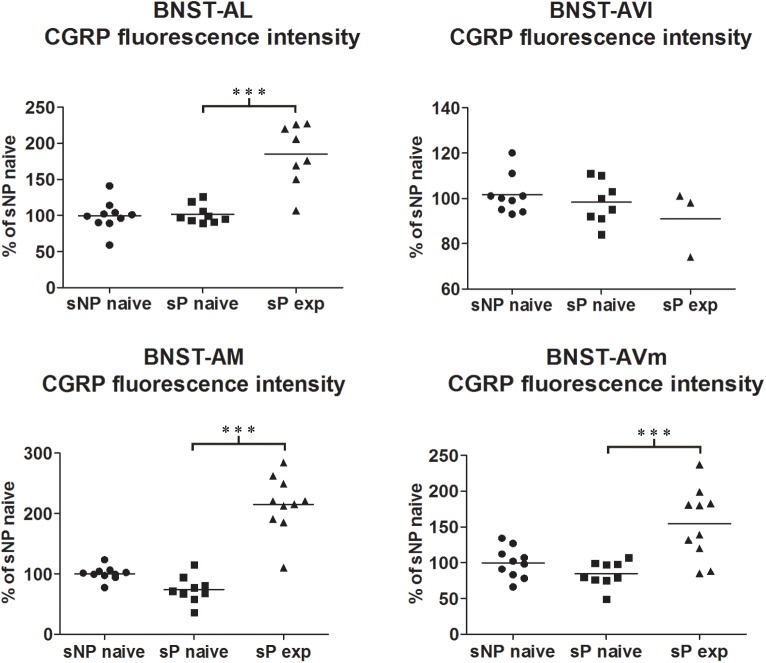
CGRP immunofluorescence intensity increased in specific BNST subnuclei following voluntary alcohol intake in sP rats. The median CGRP immunofluorescence intensity was evaluated by quantitative automatic analysis. CGRP intensity values in alcohol-naive sP rats were 97% in BNST-AL (*p* > 0.05), 98% in BNST-AVl (*p* > 0.05), 71% in BNST-AM (*p* > 0.05), and 80% in BNST-AVm (*p* > 0.05), in comparison to alcohol-naive sNP rats. In alcohol-experienced sP rats fluorescence intensity was 198% in BNST-AL (*p* < 0.001), 100% in BNST-AVl (*p* > 0.05), 307% in BNST-AM (*p* < 0.001) and 200% in BNST-AVm (*p* < 0.001) in comparison to alcohol-naive sP rats. BNST, bed nucleus of stria terminalis; AL, anterolateral; AVl, anteroventral-lateral aspect; AM, anteromedial; AVm, anteroventral-medial aspect; exp, alcohol-experienced. ^∗∗∗^*p* < 0.001.

**FIGURE 2:**
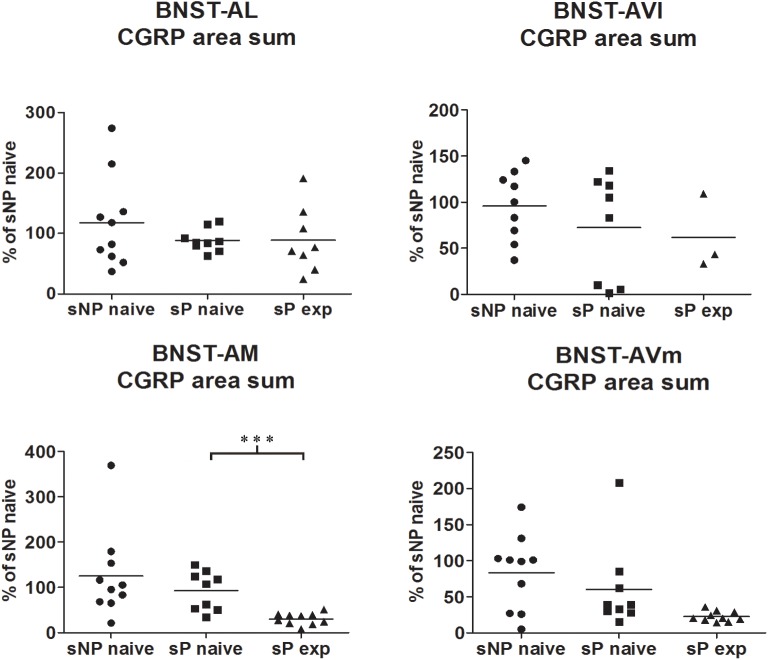
The total CGRP-labeled areas decreased in BNST-AM (but not other subnuclei) following voluntary alcohol intake in sP rats. The sum of areas of CGRP immunofluorescence within individual subnuclei was evaluated by quantitative automatic analysis. The sum of areas of CGRP immunofluorescence within individual subnuclei in alcohol-naive sP rats was 85% in BNST-AL (*p* > 0.05), 94% in BNST-AVl (*p* > 0.05), 124% in BNST-AM (*p* > 0.05), 152% in BNST-AVm (*p* > 0.05) of values of alcohol-naive sNP rats. In alcohol-experienced sP rats, area sum showed a decrease in BNST-AM (to 26% of alcohol-naive sP rats: *p* < 0.001), whereas no statistically significant differences were found in BNST-AL, BNST-AVl, and BNST-AVm (to 88, 46, and 13%, respectively, in comparison to alcohol-naive sP rats: *p* > 0.05 in all these subnuclei). BNST, bed nucleus of stria terminalis; AL, anterolateral; AVl, anteroventral-lateral aspect; AM, anteromedial; AVm, anteroventral-medial aspect; exp, alcohol-experienced. ^∗∗∗^*p* < 0.001.

However, Bonferroni/Dunn’s multiple tests revealed no differences in direct comparisons between alcohol-naive sNP and sP rats in any of the four above-mentioned BNST subnuclei, neither in CGRP-labeled fiber/terminal fluorescence intensity nor their sum of areas (see Figure 1 for results of fluorescence intensity, and Figure 2 for results of sum of labeled areas). The same was found in all other BNST subnuclei, where CGRP innervation was much lower and irregular (not shown).

**FIGURE 3:**
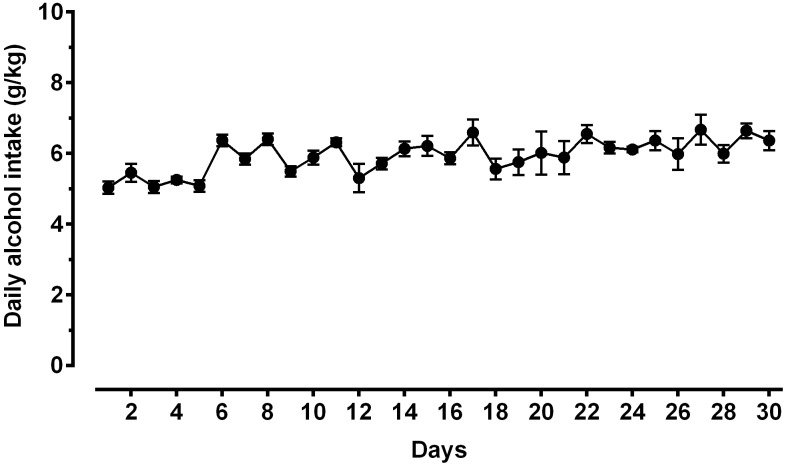
Daily alcohol intake (expressed in g/kg pure alcohol) in alcohol-experienced Sardinian alcohol-preferring (sP) rats. Rats were exposed to the standard, home-cage 2-bottle “alcohol (10% v/v) vs. water” choice regimen with unlimited access for 30 consecutive days. Each point is the mean ± SEM of *n* = 10 rats.

**FIGURE 4:**
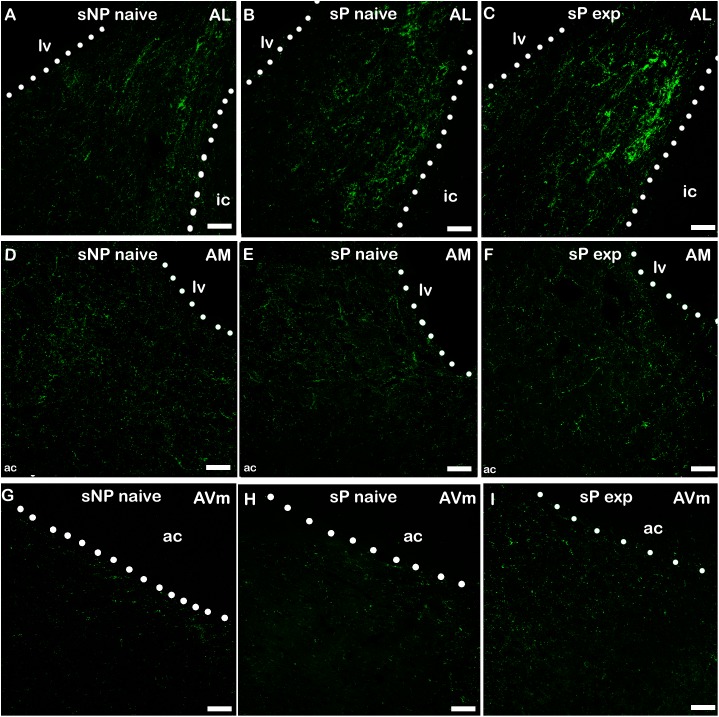
Representative examples of CGRP immunofluorescence in BNST-AL **(A–C)**, -AM **(D–F)** and AVm **(G–I)** in alcohol-naive sNP **(A,D,G)**, alcohol-naive sP **(B,E,H)**, and alcohol-experienced sP rats **(C,F,I)**. BNST, bed nucleus of stria terminalis; AL, anterolateral; AM, anteromedial; AVm, anteroventral-medial aspect; exp, alcohol-experienced; lv, lateral ventricle; ic, internal capsule; ac, anterior commissure. Calibration bar = 30 μm.

A second direct comparison was performed between alcohol-naive and -experienced sP rats. Mean daily alcohol intake in alcohol-experienced sP rats was higher than 5 g/kg from the first days of exposure to the 2-bottle choice regimen, then progressively rose on continuing exposure, and finally stabilized around 6.5 g/kg [*F*(29,261) = 3.16, *p* < 0.05] (Figure 3), replicating data repeatedly recorded in alcohol-experienced sP rats (Colombo et al., 2006). Alcohol intake strongly increased CGRP immunofluorescence intensity in BNST, in a subnucleus-specific manner. Fluorescence intensity was increased in BNST-AL, BNST-AM, and BNST-AVm of alcohol-experienced sP rats (Figure 1). In contrast, CGRP intensity value was unchanged in BNST-AVl (Figure 1). Strikingly, CGRP immunofluorescence area sum showed a decrease in BNST-AM of alcohol-naive sP rats, whereas only a non-significant tendency to decrease or no change was detected in BNST-AL, BNST-AVl, and BNST-AVm of alcohol-naive sP rats (Figure 2). Representative examples of CGRP innervation in BNST-AL, BNST-AM, and BNST-AVm in the three different experimental groups are shown in Figure 4.

### NPY Expression

NPY content was significantly different in an overall comparison among alcohol-naive sNP, -naive sP, and -experienced sP rats in all subnuclei (BNST-AL NPY intensity: *H* = 9.378, *p* = 0.0092; BNST-AL NPY area sum: *H* = 0.3178, *p* = 0.8531; BNST-AVl NPY intensity: *F* = 4.055, *p* = 0.0332; BNST-AVl NPY area sum: *H* = 3.497, *p* = 0.1740; BNST-AM NPY intensity: *F* = 6.564, *p* = 0.0056; BNST-AM NPY area sum: *F* = 6.200, *p* = 0.0070; BNST-AVm NPY intensity: *F* = 10.47, *p* = 0.0005; BNST-AVm NPY area sum: *H* = 4.456, *p* = 0.1077).

**FIGURE 5:**
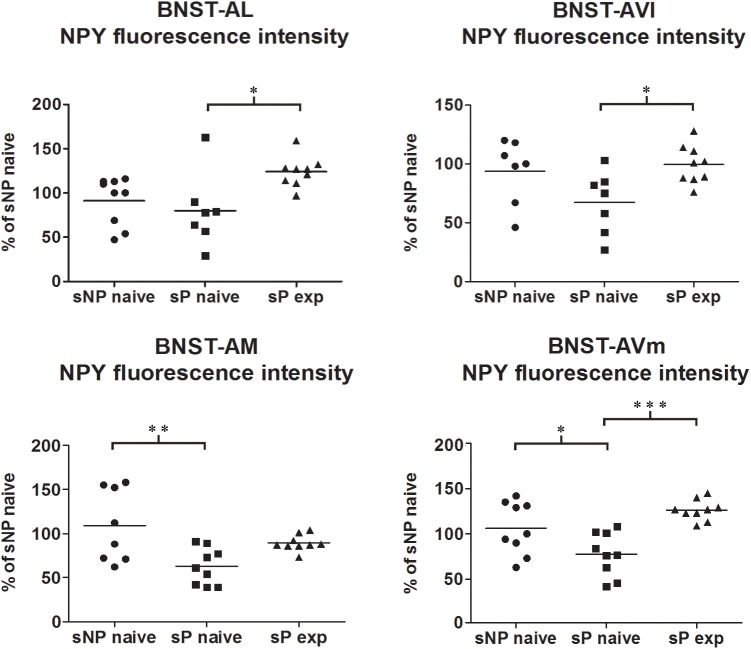
NPY immunofluorescence intensity was lower in alcohol-naive sP rats (in comparison to sNP rats) while alcohol experience increased it, in a subnucleus-specific manner. The median NPY immunofluorescence intensity was evaluated by quantitative automatic analysis. NPY intensity values in alcohol-naive sP rats were lower in BNST-AM (58%: *p* < 0.01) and BNST-AVm (77%: *p* < 0.05) than in alcohol-naive sNP rats, whereas they were unchanged in BNST-AL (71%: *p* > 0.05) and BNST-AVl (67%: *p* > 0.05). In alcohol-experienced sP rats BNST-AL, BNST-AVl and BNST-AVm showed a significant increase in fluorescence intensity (to 179%: *p* < 0.05; 152%: *p* < 0.05; 165%: *p* < 0.001, respectively) in comparison to alcohol-naive sP rats, whereas it was unchanged (although with a tendency to increase) in BNST-AM (150%: *p* > 0.05). BNST, bed nucleus of stria terminalis; AL, anterolateral; AVl, anteroventral-lateral aspect; AM, anteromedial; AVm, anteroventral-medial aspect; exp, alcohol-experienced. ^∗^*p* < 0.05, ^∗∗^*p* < 0.01, ^∗∗∗^*p* < 0.001.

Direct comparison between alcohol-naive sP and sNP rats showed that NPY fluorescence intensity was significantly lower in BNST-AM and BNST-AVm of sP rats, whereas it was not different in the other two subnuclei (Figure 5). Instead, the sum of NPY-labeled areas was not different in any subnucleus (Supplementary Figure 1).

**FIGURE 6:**
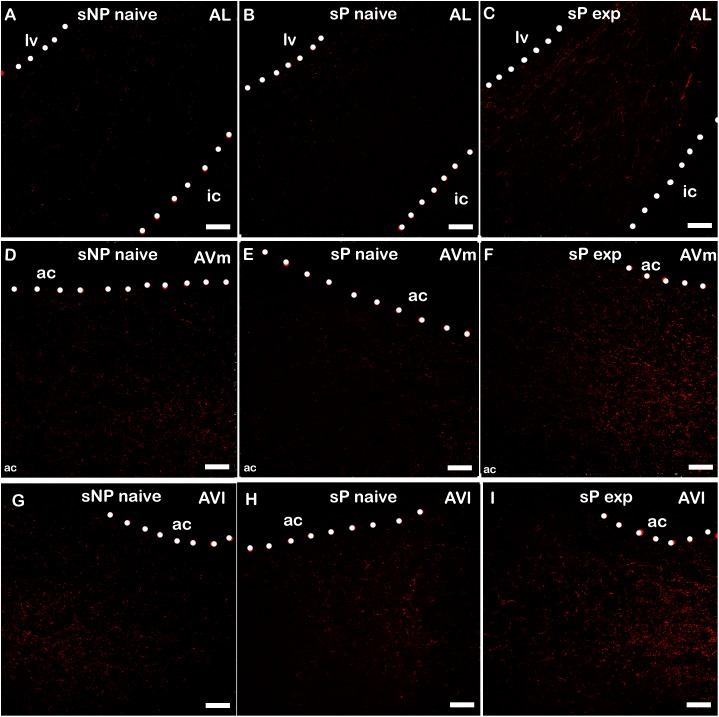
Representative examples of NPY immunofluorescence in BNST-AL **(A–C)**, -AVm **(D–F)** and AVl **(G–I)** in alcohol-naive sNP **(A,D,G)**, alcohol-naive sP **(B,E,H)**, and alcohol-experienced sP rats **(C,F,I)**. BNST, bed nucleus of stria terminalis; AL, anterolateral; AVm, anteroventral-medial aspect; AVl, anteroventral-lateral aspect; exp, alcohol-experienced; lv, lateral ventricle; ic, internal capsule; ac, anterior commissure. Calibration bar = 30 μm.

Alcohol intake changed the pattern of NPY expression, again in a subnucleus-specific manner. BNST-AL, BNST-AVl, and BNST-AVm showed a significant increase in fluorescence intensity in alcohol-experienced sP rats (Figure 5). A non-significant tendency toward an increase was observed in BNST-AM of alcohol-experienced sP rats (Figure 5). No changes between alcohol-naive and -experienced sP rats were detected in area sum of NPY-labeled areas in any of BNST subnuclei (Supplementary Figure 1). Representative examples of NPY-labeled fibers/terminals in BNST-AL, BNST-AVm and BNST-AVl are shown in Figure 6.

### Microglia Activation

Microglia activation is considered a hallmark of neuroinflammation (see, e.g., Tang and Le, 2016). Here it was analyzed by means of morphological tools that measured size and shape of Tomato Lectin-labeled microglial cells (Sardi et al., 2014; Rossetti et al., 2018). A comparison of microglia activation expression showed that many morphological parameters were significantly different among the three rat groups (alcohol-naive sNP, -naive sP, and -experienced sP) in all subnuclei. In particular, four such parameters were found to vary more consistently in stained microglia (here referred to as particle) of the three groups: mean area, major (the longest axis of the best fit ellipse), height (the longest dimension of the smallest bounding rectangle of labeled particle), minferet (the minimum caliper diameter: the minimum distance between two parallel tangents touching the particle outline in all directions). Overall analysis of mean area (by ANOVA, *F*, or Kruskall-Wallis, *H*, statistics, depending on distribution type) among the three groups provided the following results: in BNST-AL *H* = 6.061 (*p* = 0.0483), in BNST-AVl *F* = 3.966 (*p* = 0.0325), in BNST-AM *F* = 34.04 (*p* < 0.0001) and in BNST-AVm *F* = 15.91 (*p* = 0.0483). Major was found to be different in BNST-AM (*F* = 26.99, *p* < 0.0001) and BNST-AVm (*F* = 9.398, *p* = 0.0010), but not in BNST-AL (*F* = 2.597, *p* < 0.0953) and BNST-AVl (*F* = 2.870, *p* < 0.0763). Height was found to be significantly different in BNST-AL (*H* = 16.78, *p* = 0.0002), BNST-AVl (*F* = 7.039, *p* = 0.0039), BNST-AM (*H* = 16.31, *p* = 0.0003) and BNST-AVm (*F* = 8.172, *p* = 0.0020). Minferet was found to be significantly different in BNST-AL (*F* = 8.181, *p* = 0.0020), BNST-AVl (*H* = 12.71, *p* = 0.0017), BNST-AM (*F* = 32.42, *p* < 0.0001) and BNST-AVm (*H* = 14.73, *p* = 0.0006).

**FIGURE 7:**
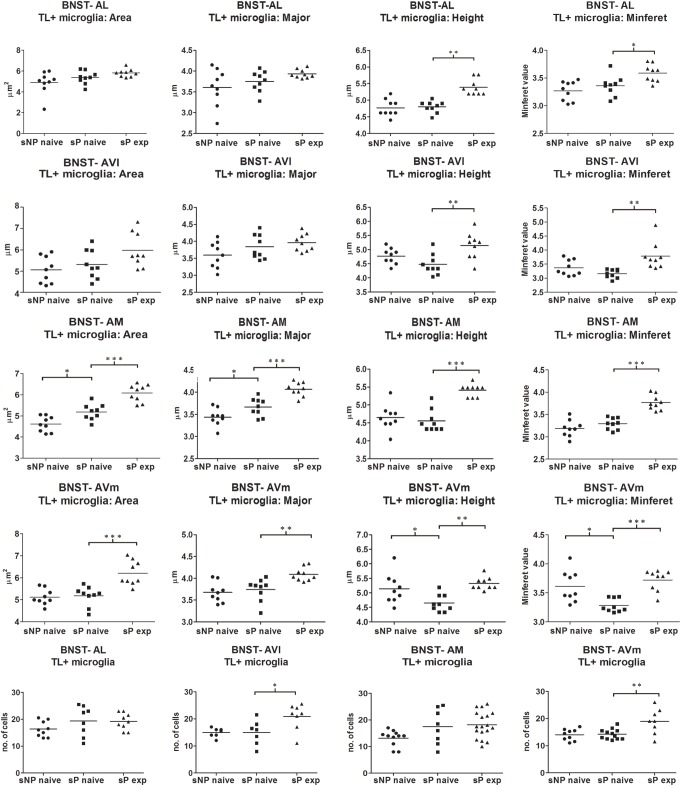
Microglia morphology (which is altered following activation) and proliferation were differentially regulated in a subnucleus-specific manner in BNST of alcohol-naive sP, alcohol-naive sNP, and alcohol-experienced sP rats. Increases in the present morphological parameters indicate microglia de-activation, whereas decreases represent a sign of activation. A quantitative automatic morphological analysis measured size and shape of Tomato Lectin-labeled microglial cells. Four parameters are illustrated here: mean Area (of labeled particle, i.e., microglia cell displaying the presence of DAPI-stained nucleus), Major (the longest axis of the best fit ellipse), Height (the longest dimension of the smallest bounding rectangle of labeled particle), Minferet (the minimum caliper diameter: the minimum distance between two parallel tangents touching the particle outline in all directions). The comparison between alcohol-naive sP and sNPrats revealed no changes in BNST-AL and BNST-AVl, whereas it revealed increases of mean area and major values in BNST-AM (114.54%: *p* < 0.05, 106.43%: *p* < 0.05, respectively), and decreases of height and minferet values in BNST-AVm (91.43%: *p* < 0.05, 92.94%: *p* < 0.05, respectively). In alcohol-experienced sPrats, height values increased to 110.45% in BNST-AL (*p* < 0.01), 121.67% in BNST-AVl (*p* < 0.01), 122.58% in BNST-AM (*p* < 0.001), 112.50% in BNST-AVm (*p* < 0.05). BNST, bed nucleus of stria terminalis; AL, anterolateral; AVl, anteroventral-lateral aspect; AM, anteromedial; AVm, anteroventral-medial aspect; exp, alcohol-experienced. ^∗^*p* < 0.05, ^∗∗^*p* < 0.01, ^∗∗∗^*p* < 0.001.

Direct comparison between alcohol-naive sNP and sP (as far as mean area as well as major, height and minferet shape parameters are concerned) is illustrated in Figure 7. Differences were detected in BNST-AM (increases in mean area and major) and BNST-AVm (decreases in height and minferet; Figure 7). No significant changes were detected in microglia density between the two alcohol-naive rat groups (Figure 7). No changes were detected in Tomato Lectin fluorescence intensity (not shown).

Voluntary alcohol consumption in sP rats caused increases in microglial mean area and shape parameters, again in a subnucleus-specific manner. BNST-AL and BNST-AVl: increases in height and minferet; BNST-AM and BNST-AVm: increases in mean area, major, height and minferet (Figure 7).

**FIGURE 8:**
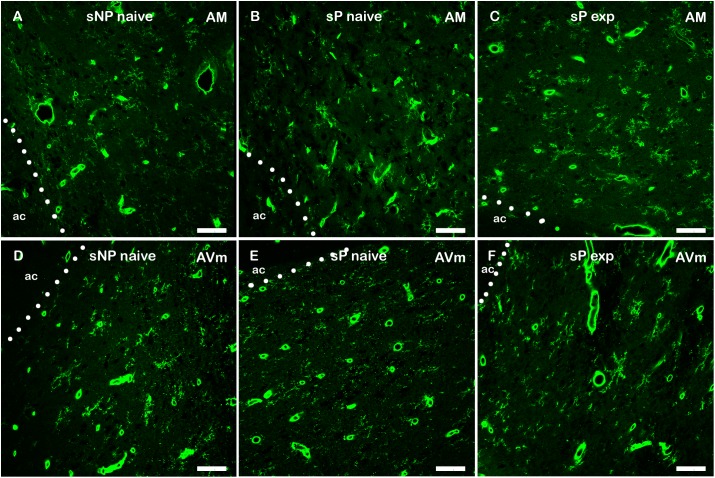
Representative examples of Tomato Lectin-labeled microglial cells in BNST-AM **(A–C)** and -AVm **(D–F)** in alcohol-naive sNP **(A,D)**, alcohol-naive sP **(B,E)**, and alcohol-experienced sP rats **(C,F)**. Tomato Lectin labeled brain vessels (saturated thick circular/elongated signal, almost invariably showing a hole in the center) and microglial cells. BNST, bed nucleus of stria terminalis; AM, anteromedial; AVm, anteroventral-medial aspect; exp, alcohol-experienced; ac, anterior commissure. Calibration bar = 20 μm.

However, a general trend toward increase was recorded in the analyzed parameters in all subnuclei (not shown). Microglia density significantly increased in BNST-AVl and BNST-AVm, whereas it was unchanged in the other two subnuclei (Figure 7). Representative examples of microglial cells in BNST-AM and BNST-AVm are shown in Figure 8.

## Discussion

Bed nucleus of stria terminalis structure is a complex collection of nuclei, with much disagreement regarding their number and location. The systematization of BNST subnuclei that was used here as framework has been described by Gungor and Paré (2016) who utilized a connectivity-dependent grouping of anterior BNST (the region mainly studied for anxiety/fear-related behaviors) in three subnuclei: (1) BNST-AL, (2) BNST-AM, and (3) AV (here split into BNST-AVl, lateral aspect, and -AVm, medial aspect). Another reference is the one produced in Swanson’s lab that is useful to compare different subnuclei classifications (Dong et al., 2001). The four subnuclei are densely connected with amygdala and hypothalamic, cortical, midbrain, and lower brainstem regions associated with emotional processing, reward, pain, and autonomic functions, and are also reciprocally connected to each other (Dong et al., 2001; Dong and Swanson, 2004; Gungor and Paré, 2016; Lebow and Chen, 2016). In particular, the main function they are thought to exert is a crucial role in anxiety-related and appetitive behaviors (Gungor and Paré, 2016; Lebow and Chen, 2016). In the present study, these nuclei showed the highest levels of CGRP, that, however, significantly increased in alcohol-experienced sP rats. In this context, it is interesting to note that sP/sNP rats (derived from Wistar rats) exhibited much lower levels of CGRP expression in BNST than Sprague-Dawley rats (not shown). In addition, an inverted ratio of CGRP expression was present between lateral vs. medial anterior BNST: Sprague-Dawley rats show high levels of CGRP expression in BNST-AL and -AVl, and low levels in BNST-AM and -AVm (see, e.g., Dobolyi et al., 2005), whereas in alcohol-naive sP and sNP rats BNST-AL and -AVl showed low CGRP levels while BNST-AM and -AVm showed moderate levels. If and how these differences are related to possible differences in alcohol consumption and/or anxiety-like states between the two rat lines remain for further investigations. Interestingly, voluntary alcohol intake inverted CGRP expression ratio between lateral vs. medial sectors in alcohol-experienced sP rats.

Although connectivity of the four subnuclei is rather overlapping, differentiating aspects are strong input from medial amygdala and subiculum to medial subnuclei (BNST-AM, -AVm), strong insular input to lateral subnuclei (BNST-AL, -AVl) as well as ventral subnuclei (BNST-AVl and -AVm) prominent noradrenergic brainstem inputs and projections to paraventricular hypothalamus and ventral tegmental area (Dong et al., 2001; Gungor and Paré, 2016; Lebow and Chen, 2016). The contextual and associative interplay mediated by extrinsic inputs is not the only determinant of the anxiety-related behavioral outcome: this depends also on the intricate asymmetric connectivity among the four subnuclei and on the balance between intrinsic GABAergic (the most abundant) and glutamatergic efferent projections (Poulin et al., 2009; Turesson et al., 2013).

sP and sNP rats differ significantly in their anxiety-related profile, with sP rats displaying inherent predisposition to anxiety-related behaviors at elevated plus maze (Colombo et al., 1995; Richter et al., 2000; Cagiano et al., 2002; Leggio et al., 2003), open-field arena (Agabio et al., 2001), and multivariate concentric square field (Roman and Colombo, 2009; Roman et al., 2012) tests. In sP rats, voluntarily consumed alcohol markedly reduced these anxiety-related behaviors (Colombo et al., 1995; Lobina et al., 2013), suggesting that anxiolysis is likely one of the central effects of alcohol that drive sP rats to seek and consume alcohol. Importantly, sP rats regulate alcohol intake to apparently maintain specific blood alcohol levels and, in turn, achieving specific central effects (Colombo et al., 2006). Here we showed that alcohol-naive sP and sNP rats differ in NPY expression in BNST. Numerous findings reported that NPY reduces alcohol intake possibly because it reduces anxiety-related behaviors and can have direct anxiolytic effects in several experimental paradigms (see Thorsell and Mathé, 2017, for a review; see, however, Pleil et al., 2015 for an indication that NPY can also have a direct effect on alcohol drinking). Thus, it can be suggested that differences in anxiety-related behaviors between sP and sNP rats can be mediated or maintained, in part, by altered NPY expression in specific BNST subnuclei.

It can be speculated that inherently determined anxiety-related behaviors of sP rats are linked also to subnucleus-specific changes in microglia activation. Neuroinflammation and microglia activation are emerging as factors promoting anxiety-related behaviors (see Sild et al., 2017; Stein et al., 2017; Kim and Jeon, 2018; Santos et al., 2018, for recent reviews). However, tissue-resident macrophages (as microglia in the brain) are also thought to constitute the first line of defense that can build up a homeostatic response directed toward repair of neural environment micro-damages in anxiety-related region (Medzhitov, 2008): this led to hypothesize that microglia activation can initially provide a beneficial support to maintain homeostasis in brain, but this error-prone response can be subsequently triggered to enter a chronic, harmful state that can lead to mental state alterations (Wager-Smith and Markou, 2011; Wohleb, 2016). The possible dual nature of microglia activation is a useful framework to interpret microglia changes in anxiety-related and appetitive behavior context: however, it seems here necessary to add further levels of complexity. One might arise from the concomitant neuropeptide expression changes, another one from subnucleus-specific connectivity: both could sum their influence to obtain the final balance of microglia activation. Indeed, it is worth mentioning that within each BNST subnucleus of alcohol-naive sP rats the significant variations of microglia morphological indices showed the same direction: the increase in mean area in BNST-AM indicates a morphological change toward a less-activated microglia, as it is the increase in major value. The same applies to the decreases of height and minferet, both indicating a morphological change toward more-activated microglia in BNST-AVm.

We have here shown that voluntary alcohol consumption, thought to exert anxiolytic effects (Colombo et al., 2006), dramatically changed the picture in BNST subnuclei. In BNST-AL a strong increase in CGRP fluorescence intensity occurred. In this region, inhibition of CGRP signaling disrupts acquisition and expression of context cued fear showing that CGRP can produce a context-dependent anxiogenic effect (Sink et al., 2013). Very recently, activation of parabrachial CGRP neurons was shown to be sufficient to establish and maintain conditioned taste aversion, which could be reproduced by stimulation of parabrachial CGRP projections into BNST-AL (Chen et al., 2018). Even though development of conditioned taste aversion to alcohol (which is dependent on an anxiety-like state; Guitton and Dudai, 2004) was not observed in sP rats (Brunetti et al., 2002), it can be hypothesized that CGRP increase in BNST-AL of alcohol-experienced sP rats is part of a mechanism activated to limit alcohol intake. The results of several previous studies suggested the existence of a central “hedonic” set-point mechanism promoting and limiting alcohol drinking in sP rats; accordingly, alcohol drinking is initially promoted until specific psychopharmacological effects are perceived, and then limited, presumably to avoid possible aversive effects that would be produced by higher doses of alcohol (Colombo et al., 2006). Also, NPY increased significantly in BNST-AL. Activation of Y1 receptor into BNST-AL reduced alcohol consumption in C57BL/6J mice exposed to the “drinking in the dark” model of binge-like alcohol drinking (Pleil et al., 2015): this effect was not accompanied by changes in anxiety-like and locomotor behaviors, suggestive of the specificity of NPY effect on binge-like alcohol drinking (Pleil et al., 2015). NPY might exert a similar curbing effect in alcohol-experienced sP rats. The actions of the two neuropeptides could be synergic in reducing alcohol intake.

Alcohol intake changes also microglia activation. In BNST-AL, as in the other subnuclei, microglia activation decreased in alcohol-experienced sP rats, as revealed by generalized increases in size and shape parameters that indicate a shift from bushy/activated toward ramified/surveying morphology (see, e.g., Sardi et al., 2014). CGRP and NPY have both been described to be able to decrease microglia activation in other experimental paradigms (Ferreira et al., 2012; Gonçalves et al., 2012; Sardi et al., 2014; Rossetti et al., 2018): their increases in sP rats (CGRP and NPY in BNST-AL and -AVm, CGRP in BNST-AM, NPY in BNST-AVL) could have thus induced decreased microglia activation in all BNST subnuclei. However, no strict parallelism can be drawn between neuropeptide expression and microglia activation: lower NPY level in BNST-AM of alcohol-naive sP rats (in comparison to sNP rats) is accompanied by reduced microglia activation. As expected, other factors play a role in microglia activation within BNST subnuclei, among which neuronal activity could be likely involved (Szepesi et al., 2018). A further level of complexity in the regulation of microglia is evidenced by their increase in number in both ventral subnuclei of anterior BNST (but not dorsal ones) of sP rats. How the subnucleus-specific increase of microglial cell number can be related to alcohol intake and anxiety-related behaviors needs further investigation.

Finally, CGRP expression underwent a distinct type of regulation in BNST-AM where alcohol intake induced an increase in CGRP fluorescence intensity which was somehow counterbalanced by a decrease in area covered by neuropeptide labeling. The CGRP fluorescence intensity increase in BNST fiber/terminals was dependent on peptide expression increase in the cell bodies of parabrachial nucleus neurons (Morara et al., in preparation), site of origin of CGRP fiber/terminals present in the anterior BNST subnuclei (Shimada et al., 1985; Dobolyi et al., 2005). However, an unexpected finding was that in BNST-AM the increase in CGRP fluorescence intensity was paralleled by a decrease in CGRP-labeled area sum: while intensity levels of individual CGRP-labeled boutons increased in BNST-AM, the sum of their number decreased in this specific subnucleus. A possible explanation is that a reduction in number of CGRP-positive fibers/terminals could have occurred in BNST-AM while the remaining CGRP-positive structures increased their neuropeptide content transported from parabrachial neurons (Morara et al., in preparation). Whatever the mechanism could be (branch pruning? branch-specific neuropeptide degradation?), it should be highlighted that it seems to occur mainly in BNST-AM, as in the other subnuclei the CGRP-labeled area sum did not change.

In conclusion, it can here be hypothesized that genetic predisposition of sP rats to high alcohol preference and consumption could be in part mediated by lower NPY expression in the medial aspect of anterior BNST and subnucleus-specific differential microglia activation.

Voluntary alcohol consumption in sP rats markedly changed CGRP and NPY expression and microglia activation in a subnucleus-specific manner. Alcohol intake significantly reduced anxiety-related behaviors (Colombo et al., 1995; Lobina et al., 2013), suggesting that anxiolysis is likely one of the alcohol effects motivating sP rats to seek and consume alcohol. Moreover, alcohol intake in sP rats is regulated so that specific blood alcohol levels are achieved and maintained, and specific central effects, including anxiolysis, are perceived (Colombo et al., 2006). A striking subnucleus specificity characterized adaptation to alcohol availability: 1) in BNST-AL both CGRP and NPY increased, and microglia de-activation occurred; 2) in BNST-AVl only NPY increased, and microglia de-activation occurred in parallel with its increase in number; 3) in BNST-AM CGRP increased in intensity, but decreased in sum of labeled areas, NPY was unchanged, and microglia de-activation occurred; 4) in BNST-AVm both CGRP and NPY increased, and microglia de-activation occurred in parallel with its increase in number. Thus, it can be here hypothesized that, in sP rats, the central effects of alcohol could be mediated, at least in part, by differential modulation of CGRP, NPY, and microglia activation/proliferation in anterior BNST, and that these changes might contribute to the anxiolytic effects of voluntarily consumed alcohol.

## Ethics Statement

This study was carried out in accordance with the recommendations of the European (Directive no. 2010/63/EU of September 22, 2010) and Italian (Legislative Decree no. 26 of March 4, 2014) laws on the “Protection of animals used for scientific purposes.” The protocol was approved by the Ethics Committee (Organismo preposto al benessere animale) of the University of Cagliari, Italy.

## Author Contributions

IR, LZ, PM, and RS performed the experimental work and contributed to acquisition and analysis of data. KG contributed to image analysis. LP, MPC, and GC critically revised the manuscript. GC and SM contributed by work design and data interpretation. SM wrote the manuscript and contributed to data and image analysis.

## Conflict of Interest Statement

The authors declare that the research was conducted in the absence of any commercial or financial relationships that could be construed as a potential conflict of interest.

## Footnotes

1 http://mosaic.mpi-cbg.de/?q=downloads/imageJ; v1.0.15

2 http://imagej.nih.gov/ij; v1.52h
