# Supplementary figures and images for: Predisposition to Alcohol Drinking and Alcohol Consumption Alter Expression of Calcitonin Gene-Related Peptide, Neuropeptide Y, and Microglia in Bed Nucleus of Stria Terminalis in a Subnucleus-Specific Manner
Source: Front Cell Neurosci. 2019 Apr 30;13:158. doi: 10.3389/fncel.2019.00158 (PMC6502997; doi:10.3389/fncel.2019.00158)

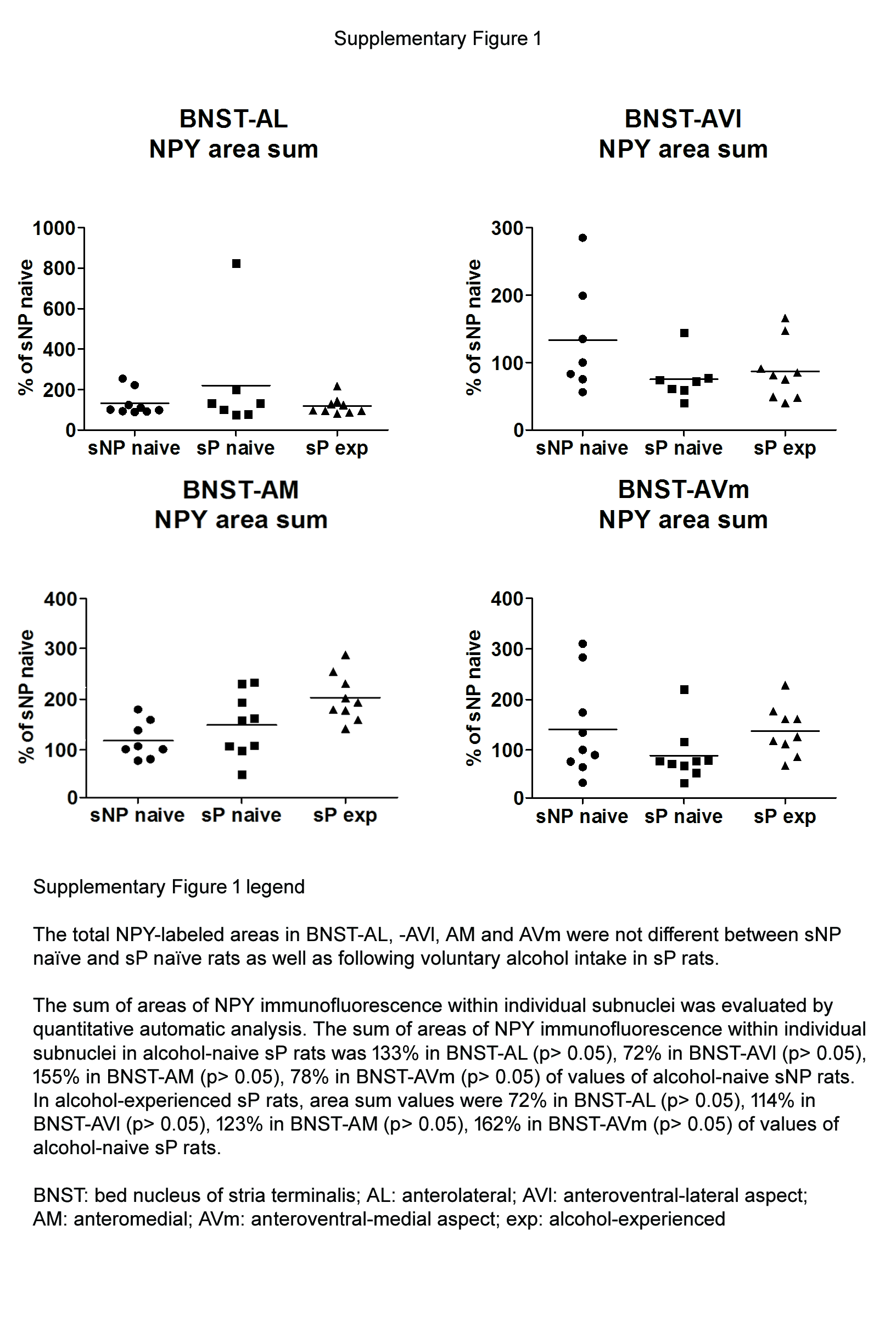

Supplement: Supplementary file 1 [file Image_1.TIF]
